# Intervention Program for Social Isolation: The Waigaya Project’s Lifelong Education Approach

**DOI:** 10.1093/geroni/igaf122.3112

**Published:** 2025-12-31

**Authors:** Keiko Katagiri, Nahyun Kim, Madoka Ogawa, Chiharu Miura

**Affiliations:** Kobe University, Kobe, Hyogo, Japan; Kobe University, Kobe, Hyogo, Japan; Osaka University, Osaka, Osaka, Japan; Advanced Research Center for Well-being of Kobe University, Ashiya, Hyogo, Japan

## Abstract

In Japan, social isolation and loneliness among older adults have become serious social issues. The purpose of this study is to examines the effects of the Waigaya Project, a lifelong education intervention program launched in 2023 to address these challenges among residents in urban rental apartment complexes, where interpersonal indifference is more pronounced. A mail survey was conducted in January 2025, targeting all residents aged 18 and older in a rental apartment complex in Kobe. A series of Analysis of Covariance (ANCOVA) were performed, with program participation as the independent variable. Dependent variables included the number of weak and strong social ties, sense of belonging, and loneliness. Control variables encompassed sex, education, years of residence, age, parental status, marital status, health and financial status, single-household status, and employment status. The results demonstrated significant positive effects of the intervention of this action research. Participants exhibited increased social ties, a stronger sense of belonging, and lower levels of loneliness than non-participants, with greater effects among those who participated more frequently. The program covered various topics, such as disaster preparedness, financial planning, well-being and generative AI. Lectures were followed by small-group discussions to enhance participant interaction. While most lifelong education programs in Japan rely solely on lectures, our findings emphasize the importance of interactive program design. This action research incorporated diverse data sources, such as real-world data from ICT devices and interviews. Further analysis is required to integrate these data and examine how the intervention reduced loneliness and what factors influenced participation.

